# Evolution and functional cross‐talk of protein post‐translational modifications

**DOI:** 10.1002/msb.201304521

**Published:** 2013-12-20

**Authors:** Pedro Beltrao, Peer Bork, Nevan J. Krogan, Vera van Noort

**Affiliations:** ^1^ European Molecular Biology LaboratoryEuropean Bioinformatics Institute (EMBL‐EBI)CambridgeUK; ^2^ Structural and Computational Biology UnitEuropean Molecular Biology LaboratoryHeidelbergGermany; ^3^ Max‐Delbruck‐Centre for Molecular MedicineBerlin‐BuchGermany; ^4^ Department of Cellular and Molecular PharmacologyUniversity of CaliforniaSan FranciscoCaliforniaUSA; ^5^ California Institute for Quantitative BiosciencesSan FranciscoCaliforniaUSA; ^6^ J. David Gladstone InstitutesSan FranciscoCaliforniaUSA

**Keywords:** acetylation, evolution, phosphorylation, post‐translational modifications, PTM cross‐talk

## Abstract

Protein post‐translational modifications (PTMs) allow the cell to regulate protein activity and play a crucial role in the response to changes in external conditions or internal states. Advances in mass spectrometry now enable proteome wide characterization of PTMs and have revealed a broad functional role for a range of different types of modifications. Here we review advances in the study of the evolution and function of PTMs that were spurred by these technological improvements. We provide an overview of studies focusing on the origin and evolution of regulatory enzymes as well as the evolutionary dynamics of modification sites. Finally, we discuss different mechanisms of altering protein activity via post‐translational regulation and progress made in the large‐scale functional characterization of PTM function.

## Introduction

Cells need to constantly sense changes in internal and external conditions, some of which have to be acted on very quickly. In such cases, conditional changes can be relayed from sensors to effectors via reversible post‐translational modifications (PTMs) of proteins. The abundance of PTMs is often regulated by enzymes that can add or remove the modifications (e.g. ‘writer’ and ‘eraser’ domains, respectively) and functional consequences can be mediated by domains that recognize and bind to the modifications (e.g. ‘reader’ domains; Seet *et al*, 2006). Not only in isolation but also in coordination, PTMs can influence numerous properties of proteins including enzymatic activity, protein interactions and subcellular location. Therefore, PTMs represent an important mechanism of regulation on protein function. Post‐translational regulatory networks require fine‐tuned crosstalk of individual players to coordinate various protein states in specific cellular conditions. While protein post‐translational modification has been studied for many years in the context of cellular signaling, very little is known about how these systems evolve when species occupy new niches. The knowledge on the respective evolutionary processes and adaptive constrains can, in turn, reveal what aspects are under selection and therefore more likely to be of high functional importance.

**Figure 1:**
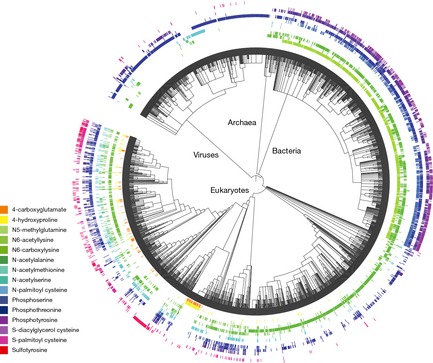
Phylogenetic distribution for the Top 15 most frequent PTMs annotated in UniProt We selected the 15 PTM types annotated in UniProt with at least 1000 occurrences and with one annotated instance in at least 10 species. For these we obtained a list of all species with at least one occurrence. The species distribution of these 15 PTM types was mapped to a phylogenetic tree using the Interactive Tree of Life tool (http://itol.embl.de/). Presence or absence of at least one occurrence is displayed using a PTM color code next to each species leaf in the tree. Absence of a PTM occurrence in any given species has to be cautiously interpreted since this could be simply due to a lack of coverage.

Recent developments in mass‐spectrometry (MS) methods now allow us to identify PTMs at an unprecedented scale (Choudhary & Mann, 2010). Thousands of PTM sites have been identified and novel enrichment strategies have uncovered the global cellular importance of several types of modifications (e.g. acetylation, ubiquitylation, *O*‐GlNac, *N*‐linked glycosylation). More than 200 different types of PTMs are currently known (Minguez *et al*, 2012), ranging from small chemical modifications (e.g. phosphorylation and acetylation) to the addition of complete proteins (e.g. ubiquitylation). Not all of these modifications have been extensively characterized. For example, 15 of these have at least 1,000 known modification sites, as annotated in Uniprot (Table 1), identified across a broad range of species (Fig 1). For a small subset of known PTM types, established enrichment protocols and mass‐spectrometry allow for the large‐scale identification of thousands of sites per study revealing important and broad functional roles (Choudhary & Mann, 2010). This increase in throughput is not only fueling a range of evolutionary studies but is also creating a bottleneck in the functional annotation and study of PTMs. We know the functional role of only a small fraction of all PTMs and functional studies cannot yet be carried out in high‐throughput. This bottleneck in the functional characterization of PTMs is reminiscent of earlier issues in the field of genomics when the dramatic increase in sequencing throughput created a need for the development of novel methods to study functional genomic elements (e.g. protein domains, transcriptional regulatory elements, transposable elements). Similarly, new methodologies are now needed to study the evolution and function of PTMs at a large‐scale and to decipher how single or combinatorial PTMs regulate cellular activities. Here we will review recent studies on these topics that were driven by this increased throughput in PTM identification.

**Table 1:** Top 15 most frequent PTMs annotated in UniProt

PTM id	Number of species	Total occurrences
Phosphoserine	1701	71744
Phosphothreonine	505	14604
Phosphotyrosine	1023	8280
*N*‐acetylalanine	347	2971
*N*‐acetylmethionine	161	1711
*N*‐acetylserine	357	1767
*N*6‐acetyllysine	664	13570
*N*‐palmitoyl cysteine	577	2056
*S*‐palmitoyl cysteine	295	2628
*N*6‐carboxylysine	1178	1600
4‐carboxyglutamate	97	1252
*N*5‐methylglutamine	776	1298
4‐hydroxyproline	115	1796
*S*‐diacylglycerol cysteine	551	1940
Sulfotyrosine	188	1015
Glycyl lysine isopeptide (with NEDD8)	13	47
Glycyl lysine isopeptide (with SUMO)	106	1234
Glycyl lysine isopeptide (with ubiquitin)	252	2815

We downloaded UniProt on 14^th^ of August 2013 which contained descriptions of 460 different post‐translational modifications, whereby modifications on different amino acids are counted individually (e.g. Phosphoserine and Phosphothreonine are two different PTMs). Of these, 218 of have been associated with at least one SwissProt entry. PTM types annotated in UniProt with at least 1,000 occurrences and with one annotated instance in at least 10 species (15 different PTM types) are listed here along with total counts for number of species and occurrences. Given their well‐studied functional importance, we also added information on Ubiquitin, SUMO and NEDD.

### Evolution of protein post‐translational regulation

**Figure 2:**
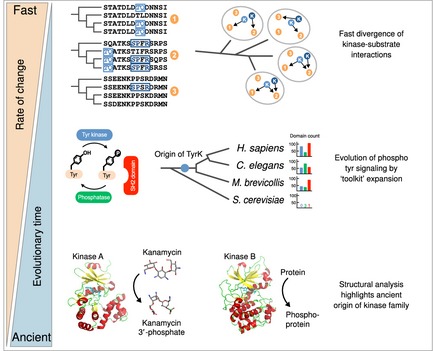
Evolution of PTM regulation at different time scales PTM evolution can be studied at different levels: from the origin of the PTMs and their enzymes (bottom) to the evolution of PTM enzymes and binding domains (middle) and their interactions (top). Many PTM types appear to be ancient and the origin of novel PTM type and their regulatory enzymes is likely to be a rare evolutionary event. The study of the ancient origin of protein phosphorylation has been aided by structural analysis where the conserved structural motifs of different phosphorylation enzymes can shed light on the origin of these enzymes. This is illustrated by the structural similarity between an aminoglycoside phosphotransferase (Kinase A, PDB: 1J7U) and a cAMP‐dependent protein kinase (Kinase B, PDB: 1CDK). Once a PTM type is established, the evolution of its regulators is likely to progress via gene‐duplication and divergence. Given the functional inter‐dependencies of the different effector domains (e.g. reader, writer and eraser) a high degree of co‐evolution is expected. This was observed for the phospho‐tyrosine effector domains (middle) where the three domain types typically display an all‐or‐none pattern of occurrence in the proteomes. The divergence of domain‐site interactions can occur at a faster rate than the divergence of effector domains since only a few mutations are required to create or destroy a PTM site. A hypothetical case illustrates how a few point mutations would be sufficient to significantly re‐wire a kinase‐substrate interaction network composed of three proteins and two kinases (top).

The evolution of post‐translational regulation, and more specifically post‐translational modification, can be studied at multiple levels (Fig 2). On a very long timescale, PTMs can be traced to follow their origin and their diversification, starting from probably very few modification types in the last universal common ancestor (LUCA) to more than 200 known today. On an intermediate scale, the evolution of individual regulators can be dissected in the context of the enzymes that add and/or remove modifications from other proteins and the reader domains. On the shortest timescale, PTMs can be compared across species to study the conservation or divergence of individual regulatory interactions and modification sites.

#### Protein PTMs are of ancient origin

Some modifications, such as phosphorylation, acetylation and glycosylation, seem to be ubiquitous across all domains of life, suggesting that these represent ancient PTMs that were used already in the last universal common ancestor. Long believed to only play a role in eukaryotes, *N*‐glycosylation and *O*‐glycosylation have now also been demonstrated in bacteria (Abu‐Qarn *et al*, 2008). Intriguingly however, the variety of monosaccharides used for bacterial glycosylation is much wider than in eukaryotes and the evolutionary relatedness is not yet clear.

A well‐studied example of a small and ubiquitous modification is lysine acetylation. While first discovered in higher eukaryotes on histone tails, it is now becoming clear that both the species range and protein repertoire of K‐acetylation is broader (Choudhary *et al*, 2009; Zhang *et al*, 2009; Wang *et al*, 2010; Van Noort *et al*, 2012). The species for which K‐acetylation data are now available range from gram‐negative and gram‐positive bacteria to eukaryotic organisms and enzymes involved in central cellular metabolism are often targeted (Choudhary *et al*, 2009; Hayden *et al*, 2013; Kim *et al*, 2013; Lee *et al*, 2013; Wu *et al*, 2013; Zhang *et al*, 2013). The levels of acetyl‐coA can influence the levels of lysine‐acetylation in the cell as well as affect enzyme activity (Cai *et al*, 2011). Thus, the nutrient status of the cell reflected by acetyl‐coA levels may directly influence metabolic activities (Cai *et al*, 2011). Acetylation on lysines can be catalyzed by Lysine Acetyl Transferases (KATs) but can also occur non‐enzymatically (Weinert *et al*, 2013), which is one of the reasons to believe K‐acetylation is an ancient PTM. Similarly, protein phosphorylation can also occur non‐enzymatically by pyrophosphates (Saiardi *et al*, 2004).

The possibility of non‐enzymatic acetylation and phosphorylation suggests an evolutionary path, whereby, in an early evolutionary phase, the regulation of protein activity (e.g. metabolic enzymes) occurred in response to nutrient status and, in a later phase, modification enzymes evolved to actively change PTM status of target proteins. Examples of such modification enzymes are sirtuins, a family of proteins which deacetylate lysines. Interestingly, sirtuin targets include metabolic enzymes, such as, for instance, acetyl‐coA synthetase, which is activated by the deacetylation of lysine‐609 by cobB, a member of the class III type of sirtuins (Starai *et al*, 2002). Sirtuins are present in both prokaryotes and eukaryotes, supporting the notion that acetylation is an ancient PTM. In eukaryotes, sirtuins have radiated into different classes (Frye, 2000), some of which are specific to acetylated lysines but others to other acyl lysine modifications such as succinylation (Du *et al*, 2011) and myristoylation (Jiang *et al*, 2013). The nutrient status is not only reflected by acetyl‐coA but also by butyryl‐coA and propionyl‐coA. Propionyl‐coA is derived from fatty acid and amino acid catabolism, whereas butyryl‐coA is formed during beta‐oxidation of fatty acids. These coA moieties have also been discovered as modifications on histone proteins as propionyl lysine and butyryl lysine (Chen *et al*, 2007). It has therefore been hypothesized that lysine propionylation and butyrylation may also regulate cellular metabolic pathways in response to cellular physiological conditions and that these modifications might further be involved in cellular signaling pathways (Chen *et al*, 2007). For now, it is however unclear which enzymes regulate these PTMs, but it can be speculated that the activity spectrum of sirtuins might extend beyond acetylation, succinylation and myristoylation, to also include propionylation and butyrylation.

For PTMs that involve the addition of entire proteins, ubiquitylation represents the best‐studied example and it results in the addition of a large protein domain (ubiquitin) to protein lysine residues. Protein ubiquitylation is most commonly known as a PTM that targets proteins for degradation but it has been recently recognized that it also has a major regulatory role in protein homeostasis, DNA repair, signaling and protein trafficking (Mukhopadhyay & Riezman, 2007; Chen & Sun, 2009). This functional diversity has been further highlighted by the proteome‐wide characterization of human ubiquitinylation (Emanuele *et al*, 2011; Kim *et al*, 2011; Wagner *et al*, 2011). How the ubiquitylation system has evolved is not completely clear; it has been suggested that it is similar to the widespread bacterial sulfur activation and delivery system in enzymatic reaction mechanism (Hochstrasser, 2009). In particular, the activation of ubiquitin by E1 enzymes is analogous to the thiocarboxylation of C‐terminal glycines of the sulphur‐carrier proteins MoaD and ThiS (Hochstrasser, 2009). More recently, the search of ubiquitin and ubiquitin‐related domains has revealed simple versions of proteasome targeting systems in bacteria and archaea by protein conjugation of so‐called ‘prokaryotic ubiquitin‐like proteins’ (PUP) and ‘small archaeal modifier proteins’ (SAMP; Burroughs *et al*, 2012; Maupin‐Furlow, 2012). SAMPylation seems to be homologous to the ubiquitylation system with the conjugation of the ubiquitin‐like SAMP proteins performed by an E1‐like activating enzyme (Maupin‐Furlow, 2012). In contrast, PUPylation appears to be a convergent evolution for proteasomal targeting via protein conjugation that is performed via a mechanism that is not homologous to the ubiquitylation system (Festa *et al*, 2010; Poulsen *et al*, 2010; Maupin‐Furlow, 2012). Although domains that are analogous to E1 enzymes can be found in bacteria, evolutionary analysis suggests that a protein ubiquitylation system was only present in the last eukaryotic common ancestor (Koonin, 2010). Since the discovery of the ubiquitin conjugating system other evolutionary related enzyme cascades have been found to perform the conjugation of ubiquitin‐like proteins such as SUMO, NEDD8, ISG15, among others (reviewed in (Hochstrasser, 2009)).

Studying the evolutionary origin of PTM catalyzing enzymes (hereafter referred to as PTM enzymes) is particularly challenging as sequence similarity becomes less reliable with evolutionary distance. For ancient enzymes like protein kinases, structural studies can reveal conserved structural motifs and suggest an evolutionary trajectory (Fig 2, bottom; Scheeff & Bourne, 2005). Similar analyses have shed light on the evolution of different PTM enzymes including protein phosphatases (Moorhead *et al*, 2009), ubiquitin ligases (Burroughs *et al*, 2008, 2009; Grau‐Bové *et al*, 2013) and acetylases (Iyer *et al*, 2008). Such analyses have been reviewed elsewhere (Anantharaman *et al*, 2007) and provide a picture of the origin and early evolution of PTM signaling systems. Not all PTMs are likely to be as ancient in origin as the ones described above but most known PTMs have not been experimentally analyzed yet in a sufficiently systematic way across all domains of life to infer their evolutionary history. Improvements in enrichment strategies, MS sensitivity and phylogenetic coverage for different PTM types will be required to advance our understanding on the origin of novel PTMs.

#### Co‐evolution of a ‘toolkit’ of PTM writer, reader and eraser domains

The toolkit of writer, eraser and reader domains allow for specificity and spatial‐temporal control of target modification. The interplay between the three domains adds the possibility for regulated recruitment and complex non‐linear dynamics that might, for example, confer memory to signaling systems. Co‐evolution of these three domain types has been analyzed to shed more light onto their functional inter‐dependencies. In particular, the evolution of toolkit domains regulating tyrosine phosphorylation is one of the few well‐studied examples. In the context of phosphotyrosine signaling, tyrosine kinases and tyrosine phosphatases act as writers and erasers, respectively and proteins harboring Src Homology 2 (SH2) domains serve as readers that bind specifically to phosphotyrosine. Protein tyrosine kinases have extensive functions in cell‐to‐cell communication in metazoans and are thought to have been crucial for the development of multicellularity (Lim & Pawson, 2010). Two types of tyrosine kinases exist: receptor tyrosine kinases that bind ligands at extracellular domains and cytoplasmic tyrosine kinases that transmit the signals that they receive from the receptors. It has been suggested that the repertoire of metazoan cytoplasmic tyrosine kinases existed already before the split of filastereans, metazoans and choanoflagellates whereas the receptor tyrosine kinases have radiated independently in these lineages. This suggests that receptor tyrosine kinases in more primitive lineages were functioning as receivers for environmental changes and have been subsequently recruited for cell‐to‐cell communication (Suga *et al*, 2012). Tyrosine phosphatases and SH2 domains are thought to have been present in premetazoan lineages devoid of tyrosine kinases. In extant fungal model organisms, that lack dedicated tyrosine kinases, SH2 domains are not known to bind phosphotyrosine (Dengl *et al*, 2009) while the phosphatases are capable of erasing this modification (Zhan *et al*, 2000). This suggests that functional tyrosine phosphatases originated first while phosphotyrosine recognition by SH2 domains arose after the origin of tyrosine kinases (Lim & Pawson, 2010). In the absence of dedicated tyrosine kinases, it is likely that tyrosine residues can nevertheless be phosphorylated by promiscuous ser/thr kinases. Indeed, tyrosine phosphorylation in plants and fungi (Holt *et al*, 2009; Nakagami *et al*, 2010) have been shown to occur at a similar extent (~5%) as in human (Olsen *et al*, 2010; Rigbolt *et al*, 2011), even though these species do not have dedicated tyrosine kinases.

The evolution of functional reader/writer/eraser PTM toolkits provided new mechanisms to encode information and to build new signaling systems orthogonal to the other cellular regulatory systems. Thus, after the emergence of tyrosine kinases, the reader/writer/eraser system was complete and the three domains begun to expand significantly. This expansion was apparently very rapid since extant species either have no tyrosine kinases and few readers/erasers or have an expanded set of the three domains (Fig 2, middle; Pincus *et al*, 2008). This expanded toolkit might have facilitated the development of multicellularity (Pincus *et al*, 2008). The addition of a new modification type to an organism's repertoire may not only provide benefits to this species but is also associated with a cost, since it is likely to add new constraints on proteome evolution. For example, the origin of tyrosine phosphorylation and the expansion of tyrosine kinases are correlated with a decrease in the tyrosine content within proteins (Tan *et al*, 2009b). This result suggests that the amount and location of modifiable amino‐acids within the proteome might be constrained by the set of PTM enzymes used by a species.

The tyrosine phosphorylation system represents one example for which evolutionary studies could trace the origin of a novel PTM type and follow the coordinated evolution of its cognate PTM toolkit domains and of the modifiable residues in target proteins. Future work on other PTM types will be required to verify the generality of these findings.

#### Evolution of PTM toolkit proteins by duplication and divergence

Once a PTM writer/eraser/reader toolkit exists, its expansion within the proteome is expected to correlate with the evolution of substrate recognition. When a duplicate pair of these domains are retained in the genome for a large amount of time, it indicates that their function is unlikely to be redundant. Although there are many studies of specificity for writer/eraser/reader domains (Miller *et al*, 2008; Persaud *et al*, 2009; Mok *et al*, 2010), these have yet to be done within an evolutionary context. By analogy with linear‐motif interactions and TF‐promoter interactions, one might speculate that substrate recognition will follow similar evolutionary trends in the case of PTM domains. From previous studies on SH3, PDZ and TFs specificity and evolution (Tonikian *et al*, 2008; Tuch *et al*, 2008; Xin *et al*, 2013), we expect that orthologous PTM domains will often have conserved substrate recognition. Since these domains usually have many interaction partners, a change in specificity would have very drastic effects by simultaneous disrupting tens to hundreds of interactions. Therefore, specificity changes are expected to be largely driven by divergence after duplication, as it has been observed for SH3 and PDZ domains (Tonikian *et al*, 2008, 2009; Xin *et al*, 2013). It is important to note that recognition might be also determined independently from the substrate recognition site by additional factors such as localization, expression or interaction with adaptor proteins. Therefore, divergence after duplication could also be achieved by a change in these other factors. The main cell cycle kinases are an interesting example of how substrate recognition and localization work together to ensure specificity (Alexander *et al*, 2011). While the Aurora kinases and Plk1 share sub‐cellular localizations during the cell cycle, they have mutually exclusive substrate recognition. Conversely, Nek2 and Plk1 or Nek2 and the Aurora kinases have partially overlapping substrate recognition motifs but would not target the same proteins due to non‐overlapping localizations (Alexander *et al*, 2011).

Although we expect that orthologous PTM enzymes will have mostly conserved substrate recognition, exceptions have been studied in transcription‐factor recognition (Baker *et al*, 2011) and are thus possible also for PTM enzymes or binding domains.

#### Fast divergence of enzyme‐protein interactions and PTM site position

Large‐scale identification of PTMs across multiple species has opened the door to comparative studies regarding the evolutionary conservation of the modification sites, interactions and function. These studies have primarily focused on protein phosphorylation given its broad functional role and well established detection methods. Most of the comparative analyses performed to date have studied the conservation of the modified residues in alignments of orthologous proteins (Holt *et al*, 2009; Landry *et al*, 2009; Nguyen Ba & Moses, 2010; Gray & Kumar, 2011). There is some debate regarding the extent of conservation: some studies report little to no conservation of the modified residues when compared to unmodified amino‐acids (Holt *et al*, 2009; Landry *et al*, 2009; Nguyen Ba & Moses, 2010), whereas others observe a significant constraint due to the phosphorylation (Gray & Kumar, 2011). Overall, a reconciled view on these finding is that phosphorylated residues show a significant but small increase in conservation when compared to the similar unmodified residues within the same proteins when taking into account ordered and disordered regions separately. Similar conclusions were derived from the conservation of the phosphorylation state by comparing different phosphoproteomes (Beltrao *et al*, 2009, 2012; Tan *et al*, 2009a).

Two caveats regarding PTM site conservation are worth noting: mimetic amino‐acids and PTM abundance. Some amino‐acids can sometimes mimic PTMs but few studies have taken this into account in comparative analysis. For example, the negatively charged Asp/Glu can often mimic phospho‐Ser/Thr and analysis of sequence alignments of highly conserved proteins suggest that on the order of 5% of phosphosites occur in positions that likely were Asp/Glu in the ancestral state (Pearlman *et al*, 2011). These positions may highlight phosphosites that positively regulate proteins by conditionally restoring the negative charges present in the ancestral states (Pearlman *et al*, 2011). Only a fraction of the total pool of a given protein is likely to be modified at any given time but only a few studies have been able to measure PTM stoichiometry at a large scale (Wu *et al*, 2011). Using these measurements it has been shown that phosphosites that are more abundant are more likely to be conserved (Levy *et al*, 2012; Tan & Bader, 2012). It is therefore possible that a fraction of sites are experimentally correct but are *in‐vivo* ‘off‐targets’ of a PTM catalyzing enzyme.

The recent availability of data for other types of modifications has allowed for comparative studies to be extended to different PTMs beyond phosphorylation (Hagai *et al*, 2012; Zielinska *et al*, 2012). The conservation of the modification state of phosphorylation, lysine acetylation and ubiquitylation has been recently compared (Beltrao *et al*, 2012). Although lysine residues are overall more conserved than the common phospho‐acceptor residues, the constraints imposed by these two lysine modifications appear also to be significant but weak when compared to the unmodified lysines. The conservation of amino‐acid residues for 13 different PTMs has revealed some variation in the levels of constraint for different PTMs with higher conservation of carboxylation followed by *N*‐glycosylation and *C*‐glycosylation and lowest conservation of sumoylation (Minguez *et al*, 2012). Overall, many types of PTMs analyzed to date show weak evolutionary constraints in that the modified residues are only somewhat more conserved than equivalent unmodified ones.

Two justifications have been proposed for the observed low constraints due to PTMs. One possibility would be that a significant fraction of the sites are not functional and have no impact on fitness when mutated. In support of this, phosphosites with a known kinase regulator or characterized functional role show a significantly higher conservation when compared with sites from high‐throughput studies (Landry *et al*, 2009; Nguyen Ba & Moses, 2010; Beltrao *et al*, 2012). A second hypothesis is that phosphorylation sites could diverge without an impact on function through redundant intermediates. Consistent with this theory, it has been shown that the average number of phosphorylation sites per protein within functional modules (i.e. pathways or complexes) is similar across species, although different residues are modified in different species (Beltrao *et al*, 2009). Within a single protein, clusters of kinase target sites can act as a functional unit whereby the position of each site within the cluster might not be strongly constrained. This could be the case if the role of the modifications were to regulate the bulk electrostatics of a protein region (Strickfaden *et al*, 2007) or to achieve a non‐linear regulatory outcome (Kõivomägi *et al*, 2011). Phosphosites are often found in clusters within proteins (Schweiger & Linial, 2010; Christian *et al*, 2012) and the clustering of sites matching a kinase motif can serve as a predictor for kinase‐target interactions further highlighting the functional importance of these clusters (Moses *et al*, 2007a). In addition, there are well‐characterized examples where even the regulation of a crucial process like DNA re‐replication is conserved but implemented via different regulatory phosphorylation sites (Moses *et al*, 2007b; Drury & Diffley, 2009). Either of the two hypotheses described above would predict that the kinase‐protein interactions should be more conserved than the individual sites. Although this prediction has not been experimentally tested, analysis of predicted kinase networks supports this hypothesis (Tan *et al*, 2009a).

Underlying either of the two arguments proposed above is a capacity to generate novel PTM sites quickly during evolution. While novel protein‐protein interactions among protein complex subunits tend to evolve via gene‐duplication and divergence (van Dam & Snel, 2008; Pereira‐Leal *et al*, 2007), new PTM sites can be created by a few point mutations (Fig 2, top). This is possible because PTM enzyme recognition of the target modification site is often mediated by a few amino‐acids within a linear peptide stretch (i.e. linear‐motifs; Diella *et al*, 2008). In fact, it might be expected that low binding specificity (high promiscuity) should in general correlate with high divergence rates for physical interactions (Neduva & Russell, 2005; Beltrao & Serrano, 2007; Shou *et al*, 2011; Sun *et al*, 2012). Many of the evolutionary properties of post‐translational modification sites and their enzyme interactions are reminiscent of those observed for transcription factor (TF) binding sites and interactions between TFs and promoters/enhancers. TFs have also interactions that are mediated by a small number of determinant sites, which diverge quickly. Thus, similar arguments about the functional importance and co‐evolution of transcriptional interactions have been made (Moses & Landry, 2010). This analogy has been well described and allow us to generalize these evolutionary properties (e.g. divergence rates, degree of functional constraint, existence of non‐functional interactions) and their functional consequences to other interaction types and layers of regulation that are determined by degenerated sequence motifs such as splicing, miRNA‐binding sites, protein localization signals or protease cleavage sites.

### Functional importance of protein post‐translational modifications

**Figure 3:**
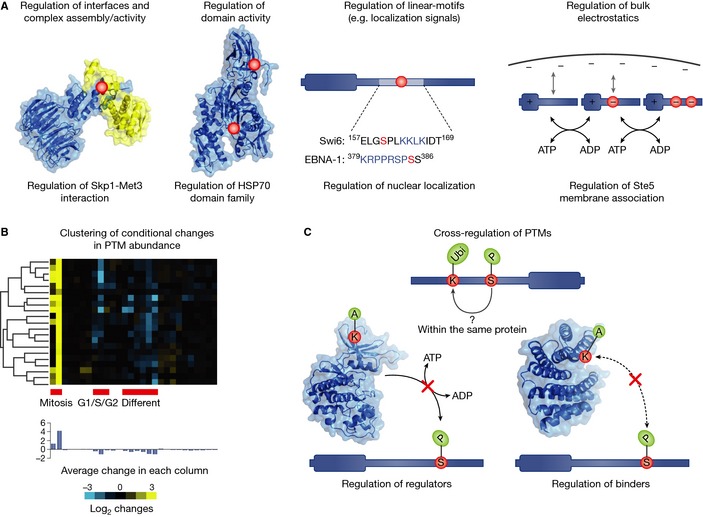
Functional role of post‐translational modifications PTMs act to change the activity of proteins through different mechanism and in response to different conditions. (A) Different mechanism used by PTMs to regulate protein activity. (B) Example of conditional regulation of phosphorylation sites. A hypothetical dataset of conditional regulation of phosphosites under different conditions was subjected to hierarchical clustering. The cluster shows a set of co‐regulated sites that is up‐regulated during mitosis and down‐regulated during stem‐cell differentiation and G1/S/G2. This cluster illustrates how patterns of co‐regulation provide additional functional annotation to PTMs. (C) Mechanism of cross‐regulation between different PTM types. Two different PTM types have been observed to cross‐regulate each other in the same protein where for example a phosphosite may recruit an E3‐ligase promoting protein ubiquitylation. The regulatory enzyme of one type can be regulated by modification of another type such as the regulation of protein kinases via acetylation (regulation of regulators). Additionally, a binder for one PTM type may be regulated by a modification site of a different type. This has been observed for 14‐3‐3 domains which can bind phosphosites and have been shown to be regulated by acetylation (regulation of regulators).

As we described above, a significant fraction of PTM sites within proteins appear to be under low evolutionary constraints and many are not conserved across species. In order to understand if these modifications are in fact not functional or if they are diverging but keeping their function through redundant evolutionary intermediates, we need to determine their functional role (Fig 3). It is possible to use the conservation of PTM sites (Budovskaya *et al*, 2005; Lam *et al*, 2010) or the conservation of predicted enzyme‐PTM interactions (Tan *et al*, 2009a) to highlight sites that are more likely functionally important. However, identifying important PTMs through conservation does not predict the function of the modifications and cannot identify species‐specific functionally important sites. In order to address this and to cope with the large amounts of PTM data being generated by mass‐spectrometry, several groups have recently tried to develop computational and experimental methods that can rank PTMs according to functional importance and assign a putative functional role.

#### Conditional regulation of PTMs

One approach to identify functionally relevant sites is to study their dynamical regulation using quantitative mass‐spectrometry approaches that can measure changes in PTM abundance under different conditions (Choudhary & Mann, 2010; Fig 3B). Sites that are regulated under specific conditions are more likely to have a functional role when compared to unregulated sites. In line with this, regulated phosphosites show an above average conservation across species (Beltrao *et al*, 2012). These quantitative experiments also place the role of the modification in the context of a specific condition, allowing for more focused experimental follow up characterization. There are several recent studies that have used quantitative approaches to find PTMs involved in different biological processes such as cell cycle (Olsen *et al*, 2010), DNA damage (Matsuoka *et al*, 2007; Bennetzen *et al*, 2010; Bensimon *et al*, 2010; Beli *et al*, 2012) and stem‐cell differentiation (Rigbolt *et al*, 2011). This concept was recently taken in an exciting research direction by integrating quantitative phosphosite information with changes in metabolic fluxes obtained under the same conditions. The correlation between these two different types of dynamical information allowed for the identification of phosphosites that regulate metabolic enzyme activity (Oliveira *et al*, 2012). Correlation of changes in PTM abundances with quantitative changes in other meaningful read‐outs (e.g. metabolic fluxes, gene expression, protein localization, genetic interactions) under different experimental conditions (Ideker & Krogan, 2012) should allow for large‐scale identification of functionally relevant modifications.

#### Regulation of activity and interactions

PTMs are known to regulate protein activity through different mechanisms, including regulating protein‐protein interactions, protein localization, degradation, cleavage or allosterically regulating enzyme activity (Fig 3A). In addition to studying dynamical regulation, several groups have developed methods that predict the mechanistic role of PTMs. For example, it was shown that the conservation of PTMs across members of a domain family could be used to predict functionally important regulatory regions (i.e. PTM regulatory hot‐spots; Beltrao *et al*, 2012). Some of these predicted regions were previously known to allosterically regulate domain activity such as the activation loop or glycine rich region of protein kinases and specific segments of the heat‐shock protein 90. PTMs found within these putative regulatory regions are therefore more likely to regulate protein activity. PTMs that might regulate inter‐domain contacts or protein‐protein interfaces have been predicted based on structural information (Nishi *et al*, 2011; Zanzoni *et al*, 2011; Beltrao *et al*, 2012). It has been shown that phosphorylation sites tend to be enriched at interface regions and that *in‐silico* mutations predict a significant impact on binding stability for about one‐third of the complexes analyzed in this way (Nishi *et al*, 2011). It was also observed that phosphosites and lysine acetylation (but not ubiquitylation) sites at interface regions show an above average conservation (Beltrao *et al*, 2012). These results suggest that PTMs are a common way by which the cell tunes the binding affinity of protein interactions and suggests approaches to identify these regulatory PTMs at a large scale. Another analysis has focused on the interplay between phosphorylation and protease cleavage (Dix *et al*, 2012). A novel MS approach was developed that could find protein cleavage events that exposed phosphosites, many of which could either promote or inhibit protease cleavage. Many other molecular mechanisms exist to regulate protein function via modifications. For example, phosphorylation is often used to regulate protein localization (Nardozzi *et al*, 2010) or protein degradation (see below). Several examples of PTMs regulating protein activity by the regulation of linear‐motifs, such as localization and cleavage peptide signals, has been recently cataloged and analyzed in the switches.ELM resource (Van Roey *et al*, 2013).

#### PTM cross‐regulation is pervasive

Different PTMs do not only function by themselves but can coordinately determine the activity and/or function of proteins in multiple ways (Fig 3C). An example of this is the interplay between phosphorylation and acetylation in the regulation of metabolic flux. As described above, phosphorylation changes in glycolytic enzymes are correlated to metabolic flux changes in yeast. However, glycolytic enzymes are acetylated in yeast as well (Henriksen *et al*, 2012) and in bacteria, changes in lysine‐acetylation patterns have been shown to redirect metabolic fluxes (Wang *et al*, 2010). The question arises whether and how these two types of modifications are coordinated. It is yet unclear if they exert changes on the same enzyme molecules or if each have their specific target proteins within the protein pool of a given enzyme. It is also not known if the phosphorylation changes cause acetylation changes or *vice versa*. In histone tails this kind of coordination between PTMs has been studied in‐depth to a point where a histone PTM code has been proposed (Jenuwein & Allis, 2001). The examples of PTM co‐regulation on histone tails (Latham & Dent, 2007) and in p53 (Brooks & Gu, 2003) have been used to suggest that most eukaryotic proteins are under the control of cooperative PTMs to determine their molecular functions and a general PTM code has been postulated (Benayoun & Veitia, 2009). The integration of multiple PTMs in such central proteins as glycolytic enzymes also suggest that co‐regulation between PTMs is very common.

#### Cross‐regulation of PTM regulators

PTMs can activate or deactivate other regulators thereby changing the PTM status of downstream proteins. Cyclin‐dependent kinases have lysines within the first 50 amino acids that are important for the coordination of adenosine 5′‐triphosphate (ATP; Choudhary *et al*, 2009). These lysines are critical for the proper functioning of the Cdk proteins, are conserved and have also been shown to be acetylated. In fact, the increase of acetylation has been shown in Cdk9 to reduce the kinase activity (Sabò *et al*, 2008). This shows that lysine acetylation has a direct role in regulating phosphorylation levels through inhibition of the kinase. Another example of regulation via acetylation involves Polypeptide GalNAc transferases (ppGalNAc‐Ts), a family of enzymes that catalyze initiation of mucin‐type *O*‐glycosylation. Specific glycosyltransferase activity of ppGalNAc‐T2 was reduced 95% by acetylation of five lysines in the catalytic core. Acetylation of lysines in the lectin domain results in alteration of the carbohydrate‐binding ability of ppGalNAc‐T2 (Zlocowski *et al*, 2011).

#### PTM cross‐talk within and across proteins

Multiple PTMs on the same protein molecule can together determine a functional outcome. For example, combinations of phophorylation, acetylation, methylation and ubiquitination determine the regulatory activity of histones. One such case involves the regulation of chromatin dynamics via cross‐talk between phosphorylation and acetylation on histone H3, carried out by the Snf1 kinase and the HAT Gcn5, respectively (Lo *et al*, 2001). More recently, evidence of cooperativity has been collected for other proteins. Phosphorylation of the transcription factor MEF2D on S444 is required for subsequent SUMOylation of K439 (Grégoire *et al*, 2006). Hydroxylation and O‐linked glycosylation seem to be cooperative PTMs as well. For instance, in O2‐dependent signaling in *Dictyostelium*, hydroxylation of Skp1 enhances its subsequent O‐linked glycosylations (Wang *et al*, 2011). In collagen proteins, there is an interplay between glycosylated residues and nearby hydroxylated prolines to determine collagen conformation and stability. (Bann & Bächinger, 2000). Coordination between different PTMs can also occur across different proteins. For example, acetylation of 14‐3‐3 disrupts binding of phosphopeptides of RAF1 and KIF1c (Choudhary *et al*, 2009). Furthermore, this cross‐talk can extend to different proteins within the nucleosome, as it has been shown that ubiquitination on histone H2B by Rad6/Bre1 is a prerequisite for methylation of histone H3 K4 and K79 by the histone methyltransferases, COMPASS and Dot1, respectively (Shukla *et al*, 2009).

Large‐scale determination of co‐regulation between PTMs remains a hard challenge. Computational analysis of different PTM types within human proteins has shown that these often co‐occur within proteins and form clusters of putative signal integration (or PTMi spots; Woodsmith *et al*, 2013). Another step in this direction was developed using the concept of correlated evolution to discover novel types of PTM co‐regulation within proteins that are not necessarily close in sequence space (Minguez *et al*, 2012). The nature of those co‐regulations will still have to be determined but it has so far indicated a vast amount of co‐regulation in proteins that has been understudied (Minguez *et al*, 2012). Large‐scale simultaneous quantification of PTM changes for two or more PTM types after perturbation or during the course of a time dependent process may be a useful approach to detect PTM cross‐talk within or across proteins. Recent studies have measured changes in phosphorylation and acetylation after DNA damage (Beli *et al*, 2012) or phosphorylation and ubiquitylation after proteasome inhibition (Swaney *et al*, 2013). In the latter case, pairs of phosphorylation and ubiquitylation sites found to increase in abundance after proteasome inhibition were indicative of potential phospho‐degron function (Swaney *et al*, 2013). Together, these studies suggest that there is an extensive and understudied degree of cross‐talk between different PTMs and substantial effort will be required to map‐out and carefully dissect these interactions.

### Summary and future challenges

Over the past 15 years, remarkable progress has been made in understanding the functional elements within genomes and their evolution. This progress has largely been made possible due to advances in sequencing technologies coupled with comparative studies. Although studies on protein function and evolution have historically preceded genome studies, large‐scale methods to study protein abundance, interactions and modifications have lagged behind genome sequencing and gene‐expression measurements. Only recently have these proteomic measurements started to reach a scale that approaches a genome‐wide level, raising interesting challenges and providing exciting research opportunities. We are now able to measure PTMs in large‐scale and these measurements brought a renewed interest in the study of PTM evolution. Although many questions remain unanswered, a broad picture is starting to emerge. The few well characterized PTMs to date appear to be, in general, very ancient and studying their origin is therefore challenging. Metabolic proteins are very commonly modified by small modifications like lysine acetylation and phosphorylation and these PTMs can be linked to the energy level of the cell. These observations suggest that acetylation and phosphorylation might have an origin that relates to energy sensing and have since then been co‐opted to other functions. Most PTM types have yet to be studied with large‐scale proteomics approaches due to the lack of enrichment strategies. Even for the types that can be studied, there is a clear need to increase the phylogenetic coverage of the species studied. As these data become available, we will have a much clearer view regarding the origin and evolution of PTM types.

The extended use of a PTM is linked to the origin of a dedicated set of writer/eraser/reader domains. The expansion of these domains with diversification of their substrate recognition are then likely driven and at the same time limited by the need to maintain specific recognition. Uri Alon and others have likened regulatory interactions to information channels that are bounded in information capacity by their binding specificity (Itzkovitz *et al*, 2006). A novel regulatory interaction, in the form of a new PTM or new TF family, would be analogous to a new information channel that the cell can use, free from interference with other channels. For example, in this context, different protein kinases are most useful if they can be sub‐divided into sub‐families with non‐overlapping substrate specificity. The extent that this subdivision is possible is limited by the kinase fold itself and perhaps to a lesser extent by the trade‐off between substrate specificity and evolvability. Even if a PTM enzyme fold was capable of recognizing substrates with a much higher specificity, improving communication fidelity, the interactions and function of this enzyme would be also less evolvable and therefore perhaps less likely to be highly duplicated during evolution. High‐throughput methods to study PTMs and the specificity of PTM enzymes will allow for these questions to be studied in detail. Specificity of interactions is determined *in‐vivo* by many factors besides domain binding specificity. For this reason, the diversification of PTM toolkit domains by duplication can be achieved also by divergence of others factors like localization or time/condition dependent expression. A great example of this type of divergence is seen for cell‐cycle kinases (Alexander *et al*, 2011).

**Figure 4:**
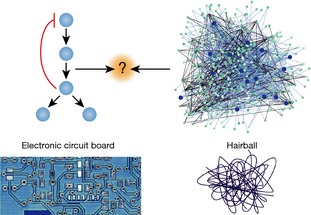
A depiction of cell‐decision making at the convergence of different approaches to cell biology Cell signaling systems are often depicted as highly logical and engineered circuits in a representation that tries to capture the main design principles of a cellular function. However, this representation may hinder our progress in studying systems that are not engineered but are highly cooperative and evolved. A second paradigm has recently been put forward, based on large scale studies of cellular networks. A view where each component is a node and each association between components is an edge. This simple network view has been useful in describing the high degree of complexity and cooperativity inside the cell but is not informative of the logic observed in these systems. We suggest that a useful and realistic description of cell biology must be informed by these two view‐points.

While new PTM types arise only rarely and PTM domain sub‐families and specificity diverge by duplication and divergence, new PTM sites and interactions have much faster evolutionary dynamics. Given the promiscuous nature of PTM toolkit domains, novel binding sites can be created in existing proteins by a few point mutations. Many PTM sites of broadly studied PTM types (phosphorylation, acetylation and ubiquitylation) identified to date are weakly constrained and are often not conserved. Additional studies will be required to increase the coverage of known PTM sites for other species and for other PTM types, as well as determining their conditional regulation and abundance. Evolutionary studies have suggested that a significant fraction of PTM sites are unlikely to have a biological role and some might change position while retaining function via redundant intermediates. These hypotheses are difficult to test experimentally and much more effort needs to be directed to the experimental study of specific signaling systems in different species and/or individuals of the same species. This view of high evolutionary plasticity of enzyme‐PTM interactions with a significant fraction of non‐functional PTMs is in stark contrast with the neatly organized signaling cascades often found in textbooks (Fig 4, electronic circuit). Signalling interactions are highly cooperative and dynamic and very often are spatially organized (Gibson, 2009). A paradigm of highly logic circuits of information cascades has initially been useful to conceptualize major signaling pathways but might also hinder our progress in a more unbiased study of signalling networks (Gibson, 2009). Large‐scale studies of cellular interactions have provided us with a different paradigm for reasoning about cell‐decision making, in which signaling components operate as part of a dense network of molecular interactions (Fig 4, hairball). This ‘nodes and edges’ network view of cell biology provides a good representation of the high degree of cooperativity between cellular components. However, this network paradigm does not convey the logic and design principles so often observed in cell biology. We suggest that an appropriate idealization of a cell must reside at the convergence of these two paradigms and will certainly be informed by evolutionary studies. Given that post‐translational and transcriptional interactions can rapidly explore novel functional space and that natural selection constrains only the emerging function and not the implementations, we expect that the same signaling function will be achieved by different species in different ways. Examples of this include the conserved timing of cell‐cycle regulation of protein complexes (Jensen *et al*, 2006), the regulation of mating (Tsong *et al*, 2006), regulation of DNA re‐replication (Kearsey & Cotterill, 2003; Moses *et al*, 2007b; Drury & Diffley, 2009) and SH3 domain function (Xin *et al*, 2013) despite changes in the underlying interactions. Comparing different implementations of crucial functions across species should highlight the important design principles underlying the function under study.

The novel high‐throughput methods to identify PTMs are creating challenges and opportunities in the functional characterization of these modifications. Novel computational and experimental methods are required to facilitate future functional studies. Some studies have attempted to achieve this by highlighting conserved PTM‐domain interactions or attempting to identify sites that have regulatory potential. Still, a clear need for additional methodologies is expected to drive research in this area. In analogy to gene‐expression studies, PTM abundance can also be measured in different conditions. The correlation of changes of PTM abundance in different conditions with measurements of other functional outputs (e.g. metabolic fluxes, protein localization, gene‐expression, protein abundance, abundance of other PTM types, etc.) under the same conditions is expected to be a powerful and large‐scale approach to identify functional PTMs. It is also becoming increasingly apparent that different PTMs tend to act in coordinated fashion. Several small‐scale studies have provided us with examples of this interplay between different PTMs and some large‐scale approaches have recently attempted to identify these functional connections on a more global scale. It is likely that many more types and instances of these cross‐regulatory interactions remain to be identified.

Taken together, this is a challenging and exciting time and analogous to the very early days of genome sequencing where tools and concepts had to build around the incoming data to transform it into knowledge. With the generation of PTM‐relevant data at large‐scale and a corresponding global analysis, we expect that many of the most exciting discoveries in the study of PTM regulation still remain ahead of us. We believe that a better understanding of the evolution and function of PTMs will result in a much more reliable picture of how these signaling proteins integrate and relay information inside the cell.
